# Kappa-Opioid Receptor Blockade Ameliorates Obesity Caused by Estrogen Withdrawal via Promotion of Energy Expenditure through mTOR Pathway

**DOI:** 10.3390/ijms23063118

**Published:** 2022-03-14

**Authors:** Amparo Romero-Picó, Marta G. Novelle, Omar Al-Massadi, Daniel Beiroa, Marta Tojo, Violeta Heras, Francisco Ruiz-Pino, Ana Senra, Miguel López, Clemence Blouet, Manuel Tena-Sempere, Rubén Nogueiras, Carlos Diéguez

**Affiliations:** 1 CIBER Fisiopatología de la Obesidad y Nutrición (CIBERobn), Instituto de Salud Carlos III, 28029 Madrid, Spain; marta.garrido@usc.es (M.G.N.); omar.al-massadi.iglesias@sergas.es (O.A.-M.); danielbeiroa@gmail.com (D.B.); violeta.heras@usc.es (V.H.); francisco.ruiz@uco.es (F.R.-P.); m.lopez@usc.es (M.L.); fi1tesem@uco.es (M.T.-S.); ruben.nogueiras@usc.es (R.N.); 2 Center for Research in Molecular Medicine and Chronic Diseases, The Center for Research in Molecular Medicine and Chronic Diseases (CiMUS), University of Santiago de Compostela, Instituto de Investigación Sanitaria (IDIS), 15782 Santiago de Compostela, Spain; marta.tojo@gmail.com (M.T.); ana.senra@usc.es (A.S.); 3 Instituto de Investigación Sanitaria de Santiago de Compostela, Complexo Hospitalario Universitario de Santiago (CHUS/SERGAS), Travesía da Choupana s/n, 15706 Santiago de Compostela, Spain; 4 Instituto Maimónides de Investigación Biomédica de Córdoba (IMIBIC), Department of Cell Biology, Physiology and Immunology, University of Cordoba, Hospital Universitario Reina Sofía, 14004 Cordoba, Spain; 5 MRC Metabolic Diseases Unit, University of Cambridge Metabolic Research Laboratories, WT-MRC Institute of Metabolic Science, University of Cambridge, Cambridge CB2 OQQ, UK; csb69@medschl.cam.ac.uk

**Keywords:** energy expenditure, estrogens, kappa-opioid, p70S6K, obesity

## Abstract

Weight gain is a hallmark of decreased estradiol (E2) levels because of menopause or following surgical ovariectomy (OVX) at younger ages. Of note, this weight gain tends to be around the abdomen, which is frequently associated with impaired metabolic homeostasis and greater cardiovascular risk in both rodents and humans. However, the molecular underpinnings and the neuronal basis for these effects remain to be elucidated. The aim of this study is to elucidate whether the kappa-opioid receptor (k-OR) system is involved in mediating body weight changes associated with E2 withdrawal. Here, we document that body weight gain induced by OVX occurs, at least partially, in a k-OR dependent manner, by modulation of energy expenditure independently of food intake as assessed in Oprk1−/−global KO mice. These effects were also observed following central pharmacological blockade of the k-OR system using the k-OR-selective antagonist PF-04455242 in wild type mice, in which we also observed a decrease in OVX-induced weight gain associated with increased UCP1 positive immunostaining in brown adipose tissue (BAT) and browning of white adipose tissue (WAT). Remarkably, the hypothalamic mTOR pathway plays an important role in regulating weight gain and adiposity in OVX mice. These findings will help to define new therapies to manage metabolic disorders associated with low/null E2 levels based on the modulation of central k-OR signaling.

## 1. Introduction

Decreased levels of estradiol (E2) in postmenopausal women or in preclinical animal models are associated with hyperphagia, reduced energy expenditure, and weight gain [1,2,3,4,5], while E2 replacement therapy prevents weight gain and metabolic dysfunctions in postmenopausal women and ovariectomized (OVX) rodents by decreasing feeding and increasing energy expenditure [3,6,7]. Increased body mass during menopause in women is characterized by a marked increase in abdominal fat, which implies a much greater cardiovascular risk [8,9]. Data gleaned over the last decade have demonstrated that by a combination of central and peripheral effects, exerted via classical estrogen receptors (α and β) and the G-protein coupled estrogen receptor (GPR30), E2 can influence energy balance, peripheral tissue insulin sensitivity, inflammation, and cardiovascular risk [3,7,10,11]. In terms of energy balance and fat distribution, estrogen receptors (ERs) are widely expressed in the hypothalamus [12], and mice with a global or brain-specific targeted disruption of ER-alpha (ERα) are obese because of hyperphagia and hypometabolism [13,14]. Interestingly, E2 displays a nucleus-specific action within the hypothalamus to modulate different compartments of energy balance and metabolic homeostasis by acting in specific hypothalamic nuclei, such as the arcuate nucleus (ARC) and the ventromedial hypothalamus (VMH) [7,14,15,16,17,18].

Similar to ER, it is known that the kappa-opioid receptor (k-OR) is widely expressed in the brain and that functional interaction between gonadal steroids and the opioid system exists [19,20], as shown by the fact that Kisspeptin/Neurokinin B/Dynorphin (KNDy) neurons located in the ARC mediate the E2-dependent decrease in body weight (BW) and increase in body temperature [21,22]. Further support of the relevant role of k-OR, in terms of energy balance, derives from the following: (i) genetic silencing of k-OR prevents high-fat diet-induced weight gain [23], (ii) k-OR -signaling is involved in ghrelin- and melanin-concentrating hormone (MCH)-induced food intake (FI) and in the anorexic effect of nicotine, as well as in its effects on energy expenditure (EE) [24,25,26], and (iii) k-OR signaling influences energy homeostasis during calorie restriction [27]. Hence, k-OR has been proposed as a promising drug target in obesity [28]. Of note, most of the studies done in relation to k-OR in energy and metabolic homeostasis were done in male animals, while their effects in females are largely unknown.

The fact that the biological effects exerted through k-OR, such as analgesia, nociception, learning, or addiction, are sex-specific and estrogen-dependent [29] make it critical to study whether k-OR is involved in mediating body weight changes associated with E2 withdrawal (e.g., in menopause or OVX), a topic that despite its relevance has not received enough attention. In this work, we disrupted k-OR signaling by genetic and pharmacological approaches to study its effects on OVX-induced weight gain. Our data show that genetic or pharmacological disruption of k-OR alleviated OVX-induced weight gain in a food-independent manner. This effect was associated with increased UCP1 immunostaining in BAT and white adipose tissue (WAT). Finally, since phosphorylated forms of mTOR/p70S6K signaling were upregulated, we addressed whether mTOR pathways could be mediating the physiological effects observed. As a result, we identified a potential mechanism based on p70S6K activation for k-OR-dependent regulation of OVX-induced adiposity.

## 2. Results

### 2.1. Genetic Deletion of k-OR Ameliorates OVX-Induced Weight Gain in Female Mice

**Figure 1:**
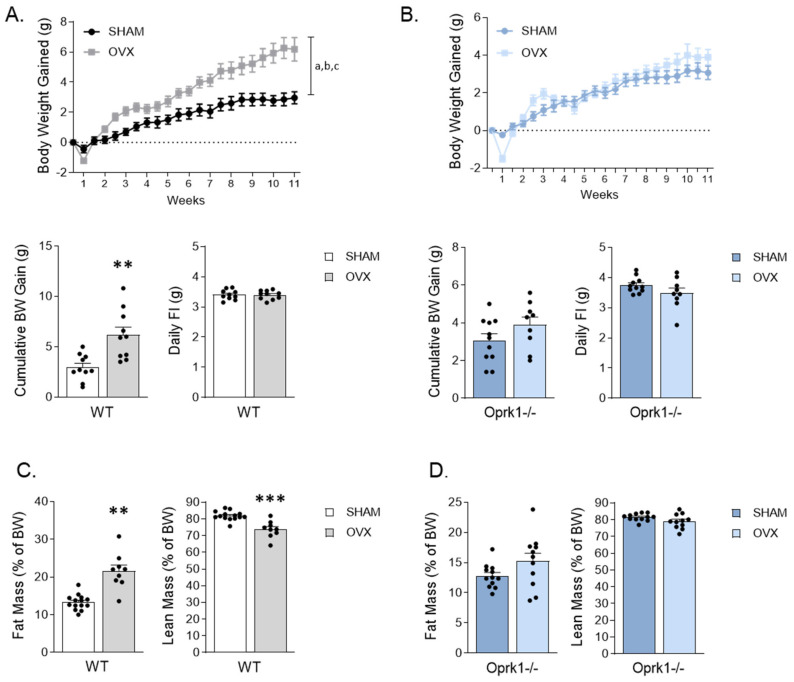
Oprk1−/−mutant mice lacking k-OR receptors are resistant to body weight (BW) gain and fat mass accumulation promoted by ovariectomy (OVX). (**A**,**B**) BW gain evolution, cumulative BW after 11 weeks and daily food intake (FI) under SHAM and OVX conditions in (**A**) WT and (**B**) Oprk1−/−mice. (**C**,**D**) results from nuclear magnetic resonance (NMR) regarding fat mass and lean mass expressed in percentage of BW in SHAM and OVX groups in WT (**C**) and Oprk1−/− (**D**) animals. Values are expressed as mean ± SEM (*n* = 8–12 per group). Two-way ANOVA (factors: OVX and time) was used to analyze BW gain evolution. Annotation indicates significant effect of a = OVX, b = time, c = interaction OVX x time. Unpaired *t*-test or Mann–Whitney test were used to compare the two groups (SHAM and OVX), indicating significant differences compared to SHAM *p* < 0.01 (**), *p* < 0.001 (***).

In keeping with previous studies [30], OVX WT mice gained more weight compared to the SHAM group (*p* < 0.01) (Figure 1A) in an FI-independent manner (Figure 1A). Interestingly, the genetic disruption of k-OR alleviated OVX-induced weight gain in a food-independent manner (Figure 1B).

In OVX WT mice, the cumulative BW gain was accompanied by an increase in fat mass (*p* < 0.01) in detriment of lean mass (*p* < 0.001) (Figure 1C), whereas this observation was absent in OVX Oprk1−/−mice (Figure 1D). These results suggest that OVX-induced adiposity and weight gain are largely mediated by k-OR signaling.

### 2.2. k-OR Animals Respond Differently to E2 Withdrawal in Terms of Circulating Luteinizing Hormone (LH) and Lipids

The absence of estrogens is characterized by elevated serum luteinizing hormone (LH). As expected in WT, LH significantly increased after 11 weeks of OVX (*p* < 0.001) compared to control group (Table 1). Similar results were obtained when we measured glucose (*p* < 0.001) and cholesterol (*p* < 0.05). Of note, basal (SHAM group) triglyceride (TG) values were lower in mutant mice than WT suggesting that lipid metabolism is affected by the absence of k-OR (Table 1).

### 2.3. Global Inhibition of k-OR Normalized the Reduced EE after OVX

**Figure 2:**
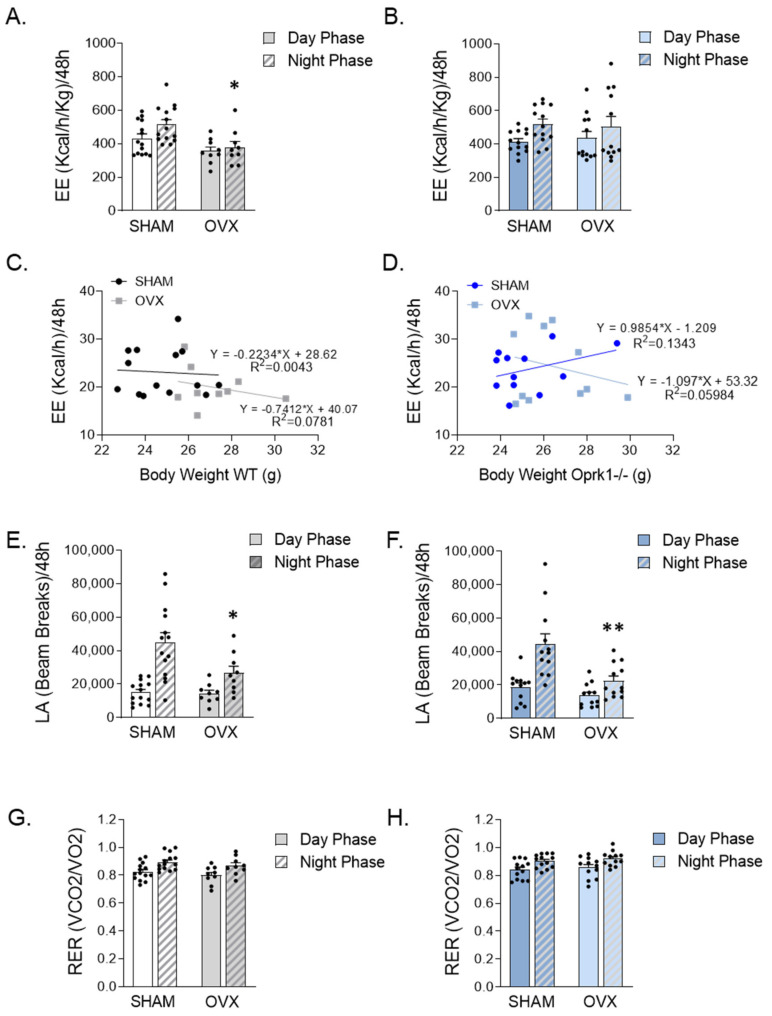
Calorimetric study indicates that Oprk1−/−mice are insensitive to EE changes induced by ovariectomy (OVX). (**A**–**D**) Total energy expenditure (EE) (Kcal/h/Kg) in (**A**) WT and (**B**) Oprk1−/−under SHAM and OVX experimental conditions in the light and dark periods during 48 h and relative to body weight (g) in WT (**C**) and Oprk1−/− (**D**) animals. (**E**,**F**) locomotor activity (LA) in (**E**) WT and (**F**) Oprk1−/−during the dark and light phases from 48 h analysis. (**G**,**H**) respiratory exchange ratio (RER) (VCO2/VO2) in (**G**) WT and (**H**) Oprk1−/−mice considering light and dark phases under SHAM and OVX conditions. Values are expressed as mean ± SEM (*n* = 8–12 per group). Unpaired *t*-test or Mann–Whitney test were used to compare the two groups (SHAM and OVX), indicating significant differences compared to SHAM (*). *p* < 0.05 (*), *p* < 0.01 (**).

To investigate the role of the k-OR system on the metabolic disturbances induced by OVX, we monitored WT and Oprk1−/−mice using indirect calorimetry chambers. We detected a reduced energy expenditure during the dark phase in WT OVX animals (*p* < 0.05) (Figure 2A,C). In contrast, no significant changes in energy expenditure were detected in the different experimental settings in mutant mice subjected to OVX (Figure 2B,D). Finally, we observed that the locomotor activity was significantly reduced during the dark phase in both WT (*p* < 0.05) and Oprk1−/−mice (*p* < 0.01) after OVX (Figure 2E,F), whereas no changes in respiratory exchange ratio (RER) were detected in WT or Oprk1−/−mice following OVX (Figure 2G,H). Taken together, these data clearly indicate that k-OR-mediated effects in weight gain following OVX are associated with energy expenditure.

### 2.4. The Role of k-OR System on BAT and WAT

**Figure 3:**
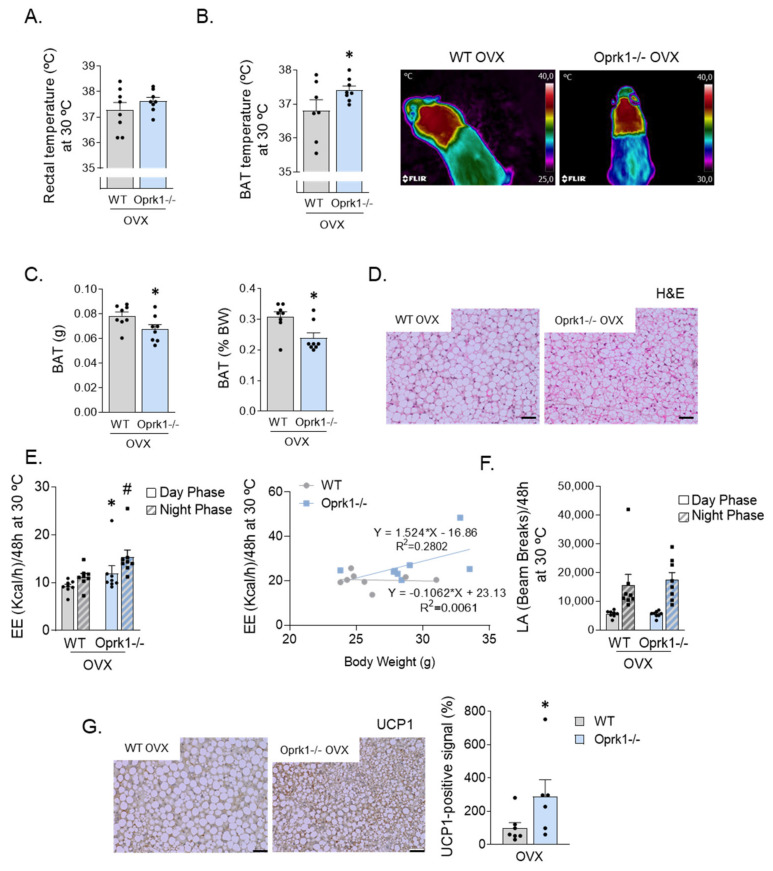
Increased thermogenic program in ovariectomized k-OR-deficient mice under thermoneutrality. (**A**) Rectal temperature at 30 °C. (**B**) Quantification of brown adipose tissue (BAT) interscapular temperature and representative infrared thermal images under thermoneutrality conditions. (**C**) Total BAT weight (g) and expressed in percentage of body weight (BW). (**D**) BAT Hematoxylin and Eosin stating (H&E). (**E**) Total energy expenditure without taking BW into account (kcal/h) in OVX-WT animals and OVX-Oprk1−/−mice and energy expenditure (EE) in WT and Oprk1−/−relative to their body weight (g). (**F**) Locomotor activity measured in beam breaks in OVX-WT and OVX-Oprk1−/−mice. (**G**) Uncoupling protein 1 (UCP1) immunostaining in OVX-WT and OVX-Oprk1−/−mice subjected to thermoneutrality. Scale bar: 20 µm. Values are expressed as mean ± SEM (*n* = 6–8 per group). *t*-test or Mann–Whitney (non-parametric conditions) was used to compare the two groups (OVX-WT and OVX-Oprk1−/−), indicating significant differences compared to OVX-WT animals. *p* < 0.05 (*) (# vs. WT night phase).

Based on the hypothesis that the lack of response to E2 withdrawal-induced metabolic changes in the global KO mice are food-independent and may be explained by increased thermogenesis, we studied in more detail BAT function and browning in an OVX mice model. We measure rectal and interscapular BAT temperature in a 23 °C (Figure S1A), cold (4 °C) (Figure S1B), and thermoneutral (30 °C) environment (Figure 3). The reason for testing at different temperatures is that at environmental temperatures below thermoneutrality, possible effects of thermogenic agents can be masked by a compensatory decrease in thermoregulatory thermogenesis, i.e., the heat production occurring in order to defend the body temperature. We found increased BAT thermogenic capacity in OVX Oprk1−/−mice since BAT temperature was higher than in OVX WT (*p* < 0.05) (Figure 3A,B) with a significant reduction in BAT mass (*p* < 0.05) (Figure 3C,D). OVX Oprk1−/−mice also displayed elevated EE (*p* < 0.05) independently of LA (Figure 3E,F) compared to OVX WT. According to the higher BAT temperature, uncoupling protein (UCP1) levels were increased in BAT of OVX Oprk1−/−mice compared to OVX WT (*p* < 0.05) (Figure 3G).

**Figure 4:**
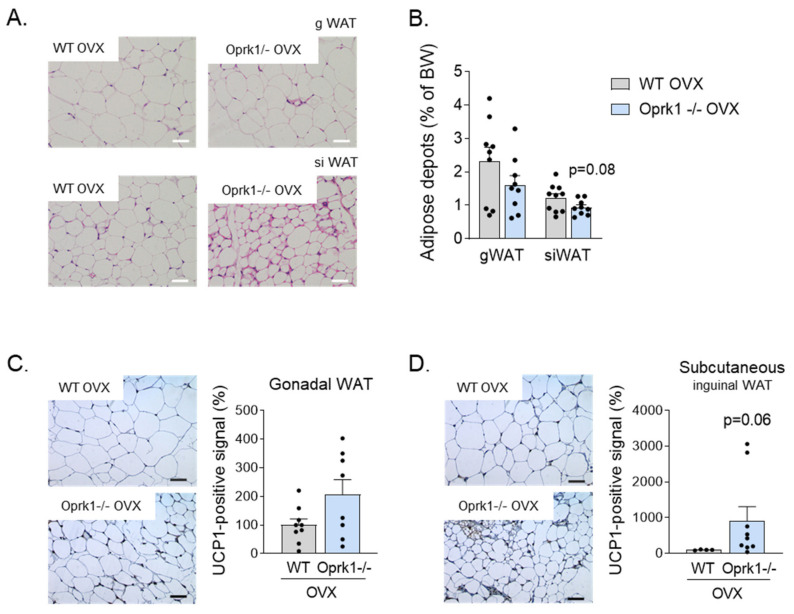
WAT browning in the E2 withdrawal model. (**A**) Representative images of Hematoxylin and Eosin staining of gonadal WAT (gWAT) and subcutaneous inguinal WAT (siWAT) from WT and Oprk1−/−mice under OVX conditions. Scale bar: 50 µm. (**B**) Gonadal and subcutaneous inguinal fat depots expressed as percentage of BW in ovariectomized WT and Oprk1−/−mice. (**C**,**D**) Representative images and quantification of UCP1 immunostaining in gWAT (C) and subcutaneous inguinal WAT (siWAT) from same animals. Values are expressed as mean ± SEM *(n* = 6–12 per group). Mann–Whitney (non-parametric conditions) was used to compare the two groups (OVX-WT and OVX-Oprk1−/−).

It is known that both activation of thermogenesis in BAT and the induction of beige/brite adipocytes in the WAT, a process known as browning, have a relevant impact on total energy balance in both rodents and humans [31,32,33,34]. We checked the browning in gonadal (gWAT) and subcutaneous inguinal (siWAT) white adipose tissue (Figure 4A) and measured fat depots after 11 weeks of OVX surgery (Figure 4B). Compared to WT mice, mice lacking a k-OR system showed a clear tendency for greater browning activity, especially in siWAT (*p* = 0.06) (Figure 4C,D) under estrogen deficiency conditions.

### 2.5. Central Pharmacological Inhibition of k-OR Recapitulates the Effect of Oprk1 Genetic Ablation

**Figure 5:**
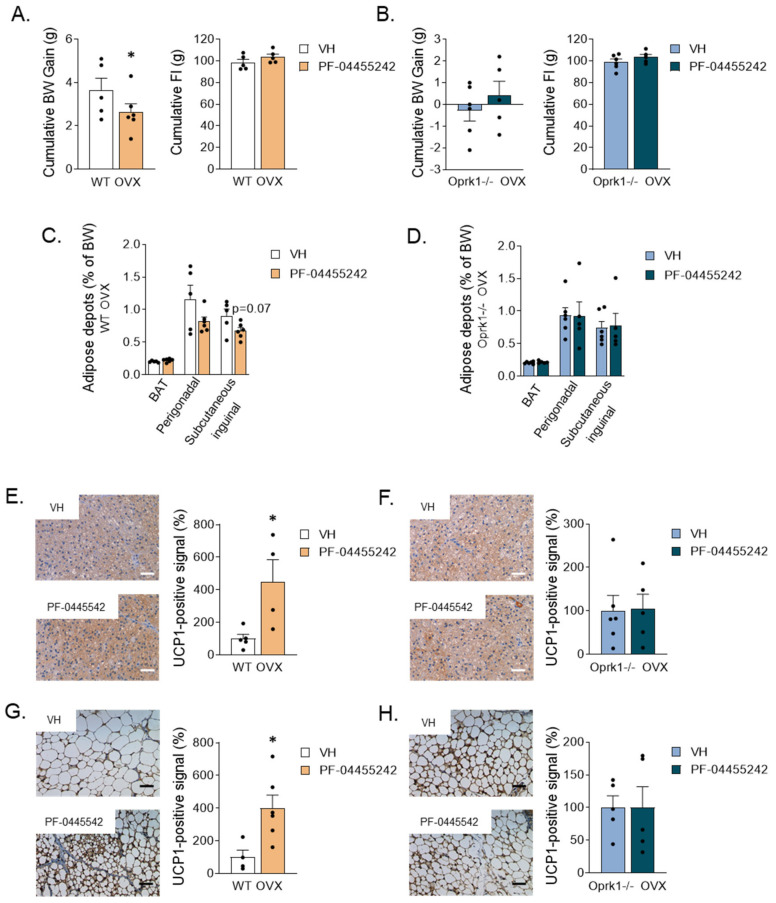
Metabolic effects of the central k-OR antagonist, PF-04455242, or vehicle (VH) administration to OVX mice. (**A**,**B**) Cumulative body weight gain and cumulative food intake monitored one week after pump brain infusion in WT (**A**) and in Oprk1−/−mice (**B**) after k-OR agonist brain infusion. (**C**,**D**) Percentage of brown adipose tissue (BAT), perigonadal, and subcutaneous inguinal fat depots (% of BW) in WT (**C**) and Oprk1−/−mice (**D**). (**E**–**H**) Representative images and quantification of uncoupling protein 1 (UCP1) levels in BAT of WT (**E**) and Oprk1−/− (**F**) animals and representative images and quantification of UCP1 protein levels in subcutaneous inguinal WAT (siWAT) in the different groups (Vehicle and PF-0445542) of WT (**G**) and Oprk1−/− (**H**) animals. Scale bar: 50 µm. Values are expressed as mean ± SEM. *t*-test or Mann–Whitney were used to compare vehicle and PF-treated mice. *p* < 0.05 (*).

Next, we chronically administrated i.c.v. PF-04455242, a specific antagonist of k-OR [35], during one week in OVX WT and Oprk1−/−mice (to discard any unspecific effects through the other opioid receptors: mu and delta). Chronic i.c.v. infusion of antagonist in OVX WT mice significantly reduced BW (*p* < 0.05) (Figure 5A), while no changes were observed in Oprk1−/−animals (Figure 5B). Of note, the reduced BW observed was independent of FI (Figure 5A,B). We noted a tendency to a reduction in siWAT depots (*p* < 0.07) after central blockade of the k-OR receptor (Figure 5C). Central administration of the k-OR antagonist also significantly increased UCP1-positive immunostaining in BAT (*p* < 0.05) (Figure 5E) and siWAT browning (*p* < 0.05) (Figure 5G) in WT mice. As expected, these k-OR antagonist-induced effects were absent in Oprk1−/−mice (Figure 5D,F,H). Non-statistically significant changes were observed in gWAT (Figure S2).

### 2.6. mTOR Signaling Mediates the k-OR-Dependent Effects on Body Weight and Adiposity in an E2-Withdrawal Model

**Figure 6:**
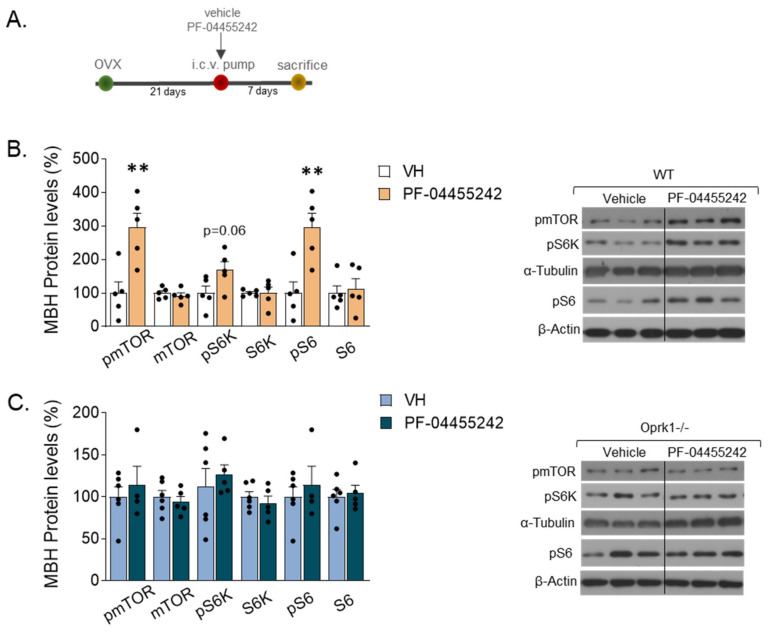
mTOR signaling pathway in the medio-basal hypothalamic area (MBH) of OVX mice after vehicle (VH) or PF-0445542 infusion. (**A**) Schematic diagram of the experimental design. (**B**,**C**) mTOR pathway analyzed by protein expression of phosphorylated and total forms of mTOR, p70S6K (S6K), and S6 after pharmacological inhibition of k-OR with the antagonist, PF-04455242, in WT (**B**) and Oprk1−/− (**C**) OVX mice (*n* = 5–6 per group). Representative blots are shown. Black line indicates cropped images. *t*-test or Mann–Whitney used to compare vehicle and PF-treated mice. *p* < 0.01 (**).

**Figure 7:**
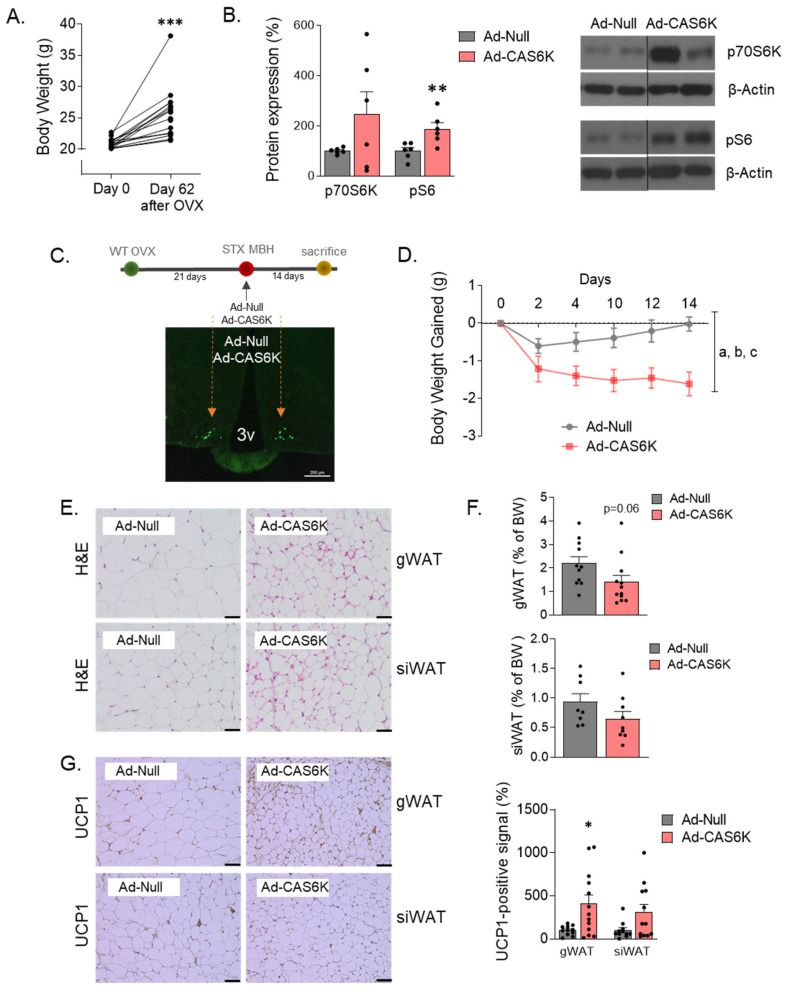
Constitutive activation of p70S6K in medial-basal hypothalamic area (MBH) reduces body weight (BW) gain induced by OVX. (**A**) Body weight (g) of WT female mice before and after 62 days of OVX surgery. (**B**) Demonstration of p70S6K constitutive activation by measurements of p70S6K and phosphorylated-S6 ribosomal (Ser240/244) protein forms in Null and CAS6K subjects. Representative blots are shown. Black line indicates cropped images. (**C**) Image depicting the stereotaxic (STX) bilateral injection of adenoviruses particles (Ad-Null or Ad-CAS6K) into the MBH. (**D**) BW gain evolution in WT-OVX mice after constitutive activation of p70S6K in MBH. (**E**) Histological images of WAT depots (Hematoxylin and Eosin staining). (**F**) Content of gWAT and siWAT expressed as percentage of BW in Ad-Null and Ad-CAS6K OVX-mice. (**G**) Activation of browning in gWAT and siWAT, as denoted by UCP1 protein levels detected by immunohistochemistry. Values are expressed as mean ± SEM (*n* = 6–12 per group). Two-way ANOVA was performed to analyze BW gain evolution (factors: p70S6K activation and time). Annotation indicates significant effect of a = p70S6K activation, b = time, c = interaction p70S6K x time). *t*-test was performed for comparisons between Ad-Null and Ad-CAS6K animals (* *p* < 0.05; ** *p* < 0.01; *p* < 0.001).

Mechanistically, we previously identified the mTOR pathway as a mediator of some of the central effects of estradiol in energy balance [18]. Thus, we studied whether it could also be involved in the k-OR-dependent mechanisms preventing OVX-induced adiposity. We found that after one week of i.c.v. administration of k-OR antagonist (PF-04455242) in WT OVX mice (Figure 6A), the mTOR signaling pathway was activated in the medio-basal hypothalamic area (MBH) since phosphorylated levels of mTOR, p70S6K, and S60 were increased (Figure 6B). Then, to evaluate the functional role of k-OR-mediated activation on thermogenesis, we expressed in the MBH a constitutively activated form of S6K (CAS6K) in OVX WT mice (Figure 7A,B). The accuracy of the viral injections was corroborated by the expression of GFP (Figure 7C). Under OVX condition, we observed a significant reduction of BW (Figure 7D) and a decrease in WAT mass and adipocyte size in gWAT and siWAT (Figure 7E,F), which was accompanied by elevated UCP1 levels, mainly in the gWAT (*p* < 0.01) (Figure 7G).

## 3. Discussion

Compelling evidence has demonstrated that ovarian hormone depletion (e.g., in conditions of OVX or menopause) induces a marked increase in weight gain, visceral adiposity, and cardiovascular risk, while estrogen replacement improves these parameters. Yet, the molecular underpinnings and neuronal basis of those actions remain largely unknown. This work demonstrates for the first time that k-OR signaling plays a key role in mediating the OVX-induced impairment of energy homeostasis. In detail, Oprk1−/−mice were resistant to weight gain and adiposity induced by OVX. This effect was independent of FI and relied on a higher EE.

Several studies have demonstrated the putative connection between the opioid system via k-OR and BAT thermogenesis since mice lacking k-OR were resistant to diet-induced obesity by increasing EE [23]. Moreover, we have recently reported that k-OR mediates nicotine-induced increases in energy expenditure [26], whereas others have documented the role of k-OR in mediating the hypothermic response to calorie restriction [27]. In this context, our current data provide novel evidence for the important role of k-OR in alleviating OVX-induced weight gain through a food-intake-independent mechanism, which prompted us to study the possible involvement of BAT-activation and browning as potential mechanisms mediating the beneficial effect of both genetic silencing and pharmacological blockade of k-OR. The influence of k-OR on WAT browning was consistent in three of the models of k-OR inhibition presented here: global KO, central pharmacological blockade, and targeted constitutive activation of p70S6K in MBH. Therefore, the induction of browning in the WAT could explain, at least to some extent, the resistance of body weight gain induced by OVX. The data obtained regarding BAT is not as clear-cut in experiments carried out below thermoneutrality. Before ruling out the involvement of BAT, we decided further explore, under thermoneutral conditions, to uncover the effect of thermogenic agents that can be masked by a compensatory decrease in thermoregulatory thermogenesis to defend body temperature. Using this approach, we found an increase in BAT temperature associated with an increase in UCP1 levels. Further studies in the context of a Scholander-type experiment are needed to further clarify this issue, including measuring the temperature dependence of the effects of ovarian function deficiency and Oprk1-silencing on food intake; body-, BAT-, and tail-temperature; temperature preference; and energy expenditure.

Data gleaned over the last few years in rodents and humans have uncovered the existence of sex differences in k-OR-system expression and function. Of note, most of the available data regarding sex differences were published in relation to pain nociception and mood; meanwhile, there is a paucity of data exploring their effects in energy homeostasis in females—hence the relevance of the current report [29,36].

Although not addressed in our study, it is tenable that the link between E2 and k-OR signaling may be associated with estrogen-responsive genes, and further studies to elucidate this question would be worthy. In fact, it is known that E2 down-regulates the prodynorphin mRNA expression in the anterior pituitary of OVX rats [37].

Data gleaned by us and others in recent years provided evidence indicating that hypothalamic AMPK in SF1 neurons in the VMH play a key role in energy balance by influencing sympathetic nervous system (SNS) activation, which leads to BAT thermogenesis and browning of WAT. Although the relevance of this mechanism is beyond any doubt and, in fact, has been shown to mediate some central effects exerted by multiple key hormones on energy balance [38], there is evidence that additional mechanisms are also involved. We provide here evidence of an alternative k-OR-dependent regulation of OVX-induced weight gain and adiposity. We documented the molecular underpinning mediating these interactions, uncovering the involvement of the mTOR-p70S6K pathway. In an E2-withdrawal model, the upregulation of phosphorylated forms of mTOR, p70S6K, and S6 under pharmacological inhibition of k-OR strongly suggest that mTOR is a k-OR downstream signaling pathway as it has been previously observed in other animal models and contexts [24,37,38]. Likewise, in an OVX model, we escape from E2 action, while inhibition of k-OR signaling or activation of p70S6K in the MBH promotes loss of BW and browning, thereby keeping with previous findings on the effects of mTOR in energy homeostasis [39,40,41]. However, recent studies support the idea that mTOR-related signaling pathways are important players for thermogenesis and browning, and the precise role of mTOR in heat production is controversial, probably due to the use of different experimental models and systems. Differences between brown and beige adipocytes lie in the distribution, origin, and UCP1 expression under non-stimulated conditions. In the basal state, beige adipocytes express a very low level of the thermogenic gene program compared to brown adipocytes. The beige cell’s capacity to switch between energy storage and energy dissipation strongly depends on the type of stimulus that it receives [39]. Based on distinctive features that brown and beige adipocytes possess, it is an interesting matter to identify potential mechanisms and regulators of these two adipocytes in energy homeostasis. In this line, our study provides evidence of k-OR/mTOR/p70S6K-mediated effects on WAT depots in an OVX mice model. Considering all these data together, this mechanism seems interesting as a drug target for diseases characterized by E2 privation.

These findings further our understanding of how OVX causes weight gain and paves the way for the search for novel specific drug targets for tackling obesity in conditions of ovarian hormone withdrawal, such as menopause, which affects a rather large fragment of the population, especially considering the lengthening of life expectancy. An obvious candidate for this could be the use of selective k-OR antagonists, an area in which recent developments make that a particularly feasible approach in disease states linked to alteration in estrogen levels [42].

## 4. Material and Methods

### 4.1. Animal Procedures

Female 8- to 10-week-old C57/BL6 wild type and Oprk1−/−mutant mice (Jackson Laboratory, Bar Harbor, ME, USA, strain name B6.129S2-Oprk1tmqKff/J) were housed under conditions of controlled temperature (23 °C) and illumination (12 h light/12 h dark cycle) [25,43]. They were allowed ad libitum access to water and standard chow (16% proteins, 60% carbohydrates, 3% fat, Harlan Research Laboratories, Indianapolis, IN, USA) under specific-pathogen-free (SPF) conditions. All animals were allowed to acclimate to their new surroundings for one week before they underwent experimental procedures or other manipulations.

Adult animals were bilaterally OVX, or SHAM operated under ketamine-xylazine anesthesia [44,45]. Care of all animals was within institutional animal care committee guidelines, and all procedures were reviewed and approved by the Ethics Committee of the University of Santiago de Compostela (protocol number 15005AE/12/FUN01/FIS02/CDG4), in accordance with EU normative for the use of experimental animals. At the end of each experimental setting, animals were killed by decapitation, and the tissues were removed rapidly, frozen immediately on dry ice, and stored at −80 °C until analysis.

#### 4.1.1. Experimental Setting 1: Effect of the Genetic Ablation of k-OR on the Metabolic Changes Induced by OVX

WT or Oprk1−/−mutant mice were distributed in two groups: (a) SHAM and (b) OVX. The surgical procedure was performed by modifying a protocol previously described in rats [44]. The BW and FI were monitored for 11 weeks. The whole-body composition was measured using nuclear magnetic resonance spectroscopy (NMR) imaging (EchoMRI, Houston, TX, USA), as previously described [43,46]. Animals were monitored in a custom 12-cage indirect calorimetry, FI, and locomotor activity (LA) monitoring system (TSE LabMaster, TSE Systems, Bad Homburg, Germany) for 72 h [47,48]. Data collected from the last 48 h were used to calculate all metabolic parameters. The tracking of metabolic parameters was carried out at basal (23 °C), cold (4 °C), and thermoneutrality (30 °C) conditions. Body temperature was recorded with a rectal probe connected to a digital thermometer (BAT-12 Microprobe-Thermometer; Physitemp; NJ, USA). The interscapular temperature was assessed and was visualized using a high-resolution infrared camera (E60bx: Compact-Infrared-Thermal-Imaging-Camera; FLIR; West Malling, Kent, UK) and analyzed with a FLIR-Tools specific software package [49].

#### 4.1.2. Experimental Setting 2: Effect of the Central Pharmacological Inhibition of k-OR on the Metabolic Changes Induced by OVX

To assess the chronic central effects of the k-OR selective antagonist, PF-04455242, WT, and Oprk1−/−animals were subjected to OVX 21 days after the surgery PF-04455242 (3.4 nmol/day) or vehicle (VH) were delivered i.c.v. via brain infusion kit 3 (1–3 mm) for 7 days through a catheter tube connecting the i.c.v. cannula to an osmotic minipump (model 1007D, Alzet Osmotic Pumps; DURECT, Cupertino, CA, USA), as previously described [43,46,50]. The mini pump was inserted in a subcutaneous pocket on the dorsal surface. The incision was closed with sutures, and mice were kept warm until full recovery. Body weight, food intake, lipid metabolism, and thermogenesis/browning were analyzed.

#### 4.1.3. Experimental Setting 3: Effect of Constitutive Activation of MBH p70S6K on OVX-Induced Adiposity

Plasmid pRK7-HA-S6K1-F5A-E389-R3A was courteously gifted by Dr. Clémence Blouet. Cloning and package in Ad5-CMV-GFP adenoviruses were performed at Viral Vector Production Unit (Universitat Autónoma de Barcelona, Barcelona, Spain). To elucidate the contribution of central mTOR signaling on OVX-induced metabolic changes, we bilaterally injected in the medio-basal hypothalamic area (MBH) null adenoviruses (Ad-Null) or adenoviruses encoding a constitutively active form (Ad-CAS6K) [24,51] to OVX mice. Twenty-one days after OVX, the two groups (*n* = 12 mice per group) were subjected to stereotaxic surgery (±0.4 mm lateral, −1.5 mm antero-posterior, and −5.8 mm dorso-ventral). We monitored the BW and chow FI for 2 weeks and, afterward, evaluated the impact of p70S6K activation on peripheral lipid metabolism.

### 4.2. Serum Measurements

Luteinizing hormone was measured by specific RIA [52]. Serum metabolic markers such as triglycerides, cholesterol, and glucose were also assayed using a specific enzymatic colorimetric protocol (SPINREACT, Girona, Spain) following the manufacturer’s instructions and expressed in mg/dL.

### 4.3. Western Blotting

Total protein extracts were obtained from BAT, siWAT, and gWAT. After quantification, 15–20 µg was resolved on SDS-PAGE gels (between 6.5 and 12% depending on protein molecular weight) and transferred to PVDF membranes as described in [24,25]. Membranes were probed with antibodies for uncoupling protein 1 (UCP1) (ab10983, Abcam, Cambridge, UK), for mTOR pathway: mTOR (SAB4501038; Sigma, Kawasaki, Japan), phospho-mTOR (SAB4504476; Sigma), p70S6K (#9202; Cell Signaling, Danvers, MA, USA), phospho-p70S6K (#9205; Cell Signaling), S6 (#2317; Cell Signaling), phospho-S6 (#2211; Cell Signaling); and internal controls: β-actin (A-5316; Sigma), α-tubulin (T-5168; Sigma). Secondary antibodies were purchased by Dako and used at dilution 1:5000 in 3% BSA in TBS-Tween 0.1%. Protein detection was performed using enhanced chemiluminescence reagent ECL (Amersham Biosciences, Little Chalfont, UK). Quantification and analysis of images were carried out by ImageJ software.

### 4.4. Histological Procedures

#### 4.4.1. Hematoxylin and Eosin Staining

Paraffin-embedded sections (BAT, gWAT, and siWAT) of 4 μm were cut with a microtome and stained using a standard Hematoxylin/Eosin Alcoholic procedure according to the manufacturer’s instructions (BioOptica, Milan, Italy). Sections were then rinsed with distilled water and dried for 30 min at 37 °C. They were then mounted with permanent (non-alcohol, non-xylene-based) mounting media [53].

#### 4.4.2. UCP1 Immunohistochemistry Detection

Tissues were fixed in formalin solution. Paraffin-embedded sections (3 µm) were dried overnight at 55–60 °C, de-paraffined with xylene, and then rehydrated. Antigenic recuperation was performed using citrate buffer 10 mM pH = 6 for 20 min at 96 °C. BAT (1:5000), gWAT, and siWAT (1:800) sections were incubated with anti-UCP1 (ab10983) overnight at 4 °C in antibody diluent (DAKO, Glostrup, Denmark, K8006). Sections were incubated with Envision FLEX/HPR secondary antibody (DAKO SM802). Visualization involved reaction with diaminobenzidine (DAB) (DAKO K3468). Quantification of UCP1 protein expression was performed using Frida software developed by Johns Hopkins University [48].

#### 4.4.3. Immunofluorescence

Female mice were anesthetized with ketamine-xylazine and perfused intracardially with saline (0.9% NaCl) followed by 4% PFA in PBS (pH 7.4). Fixed brains were immersed in 30% sucrose and 0.01% sodium azide in PBS at 4 °C for 2 days. Next, 3 sets of coronal sections (30-μm-thick) were cut in a freezing microtome Leica CM1850 UV (Wetzlar, Germany) and stored at −20 °C in cryo-protectant. One set of free-floating sections from each animal was washed in TBS 0.1 M and incubated in blocking solution (2% donkey serum + 0.3% Triton X-100) in TBS 0.1 M for 60 min. Then, sections were incubated with rabbit antibody against green fluorescent protein (GFP) (Abcam ab290) in blocking solution overnight at 4 °C. For GFP visualization, we used Cy3 donkey anti-rabbit (Jackson ImmunoResearch Labs, 711-165-162). Sections were then washed and coverslipped with Fluorogel mounting solution. Images were captured with fluorescence stereo microscopes Leica M205 and camera CCD Leica 7000 T.

### 4.5. Statistical Analysis

The results are expressed as mean values ± SEM. GraphPad Prism (8.0) software was used for the data analysis. We used unpaired *t*-test and paired *t*-test analyses in before-after studies. Two-way ANOVA was conducted to analyze BW weight gain evolution. In cases that normality and homoscedastic test were not passed, we used a non-parametric Mann–Whitney test. Data were considered statistically significant at *p* < 0.05.

## Figures and Tables

**Table 1:** Serum physiological parameters in SHAM and OVX, and female mice at 11 weeks after surgery. Values are expressed as mean ± SEM. *t*-test or Mann–Whitney test were performed to evaluate differences between groups indicating significant differences compared to SHAM (*). *p* < 0.05 (*), *p* < 0.01 (**), *p* < 0.001 (***). LH, luteinizing hormone; TG, triglycerides.

	Wild Type		Oprk1−/−	
	SHAM	OVX	SHAM	OVX
LH (ng/mL)	0.5 ± 0.1	5.3 ± 1.1 ***	0.5 ± 0.1	3.6 ± 0.9 **
TG (mg/dL)	91 ± 3.9	96.1 ± 4.8	68 ± 3.9	69.6 ± 3.9
Cholesterol (mg/dL)	85.9 ± 3.9	107.5 ± 6.3 *	86.2 ± 4.7	89.8 ± 2.5
Glucose (mg/dL)	125.8 ± 5.4	160.8 ± 4.9 ***	134.3 ± 7.6	162.1 ± 11.2 *

## Supplementary Materials

The following supporting information can be downloaded at: https://www.mdpi.com/article/10.3390/ijms23063118/s1.
