# Supplementary material for: Kappa-Opioid Receptor Blockade Ameliorates Obesity Caused by Estrogen Withdrawal via Promotion of Energy Expenditure through mTOR Pathway
Source: Int J Mol Sci. 2022 Mar 14;23(6):3118. doi: 10.3390/ijms23063118 (PMC8953356; doi:10.3390/ijms23063118)
Supplement: Supplementary file 1 [file ijms-23-03118-s001.zip › ijms-1580284-supplementary.pdf]

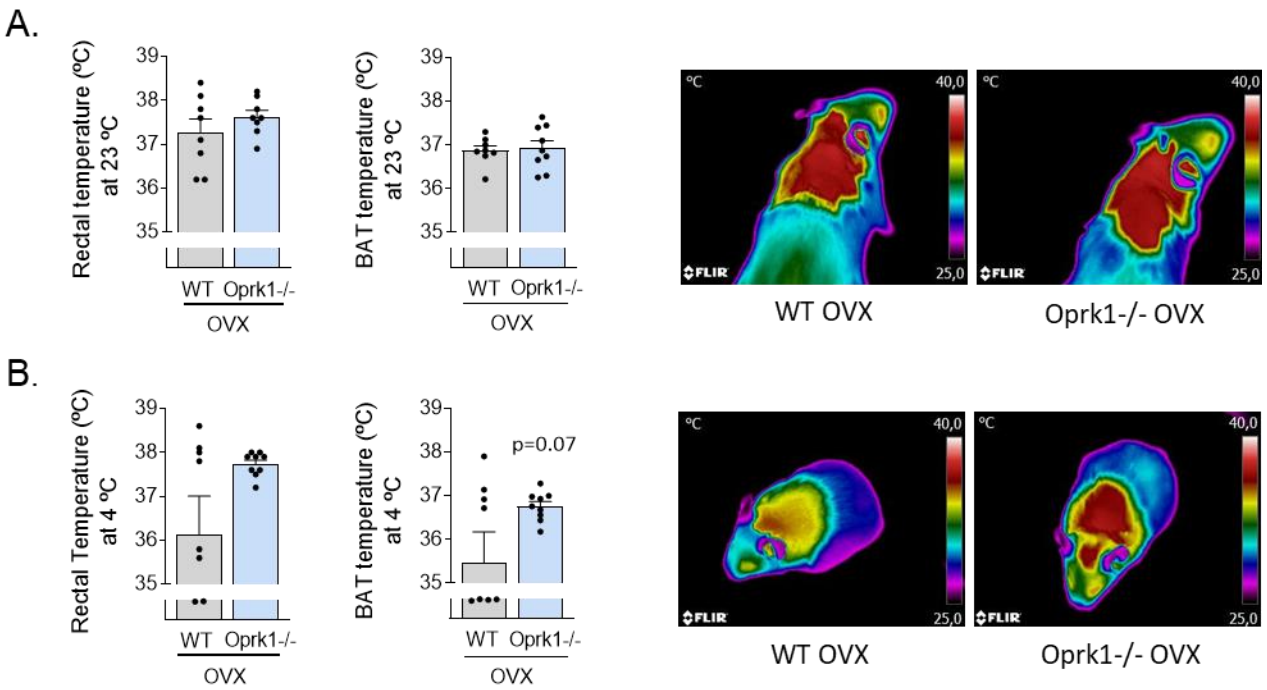

Figure S1. Corporal and interscapular temperature under basal and cold conditions. Rectal temperature, quantification of brown adipose tissue (BAT) interscapular temperature and representative infrared thermal images under 23 ºC (**A**) and 4 ºC (**B**) conditions in WT and Oprk1-/- animals subjected to ovariectomy. T-test or Mann-Whitney test were performed to compared WT and Oprk1-/- groups.

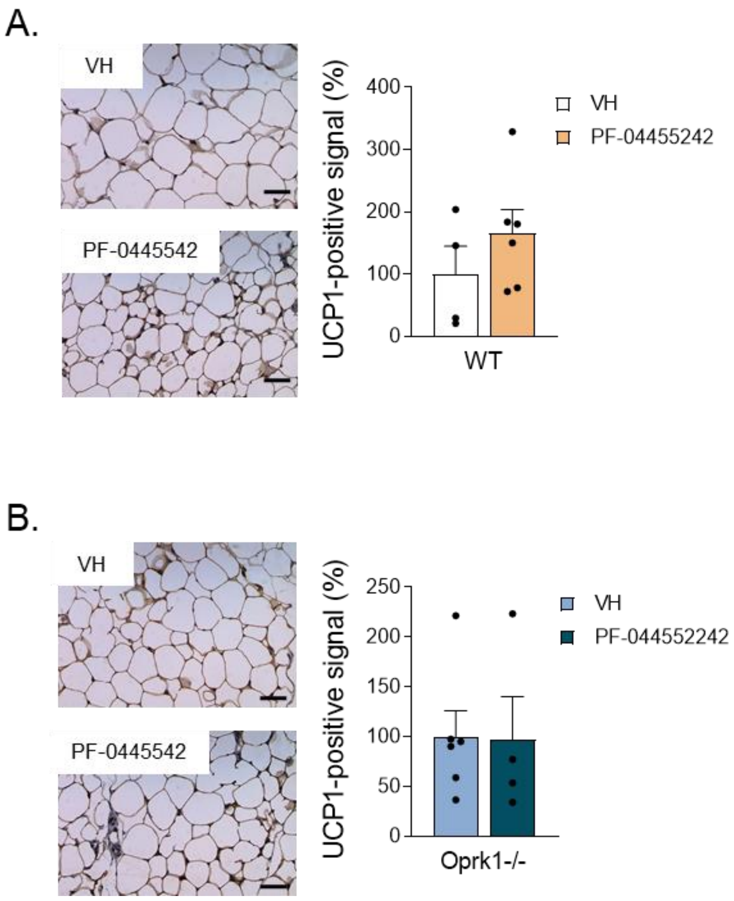

Figure S2. Effect of the central *k*-OR blockade on gonadal WAT browning. UCP1 (uncoupling protein 1) immunohistochemistry in gWAT (gonadal white adipose tissue) in both WT (**A**) and Oprk1-/- mice (**B**) after antagonizing *k*-OR with PF-0445542. Scale bar: 20µm. Values are expressed as mean ± SEM (n=5-6 *per* group). T-test or Mann-Whitney test were performed for comparisons between the two groups: vehicle (VH) *vs* PF-0445542.

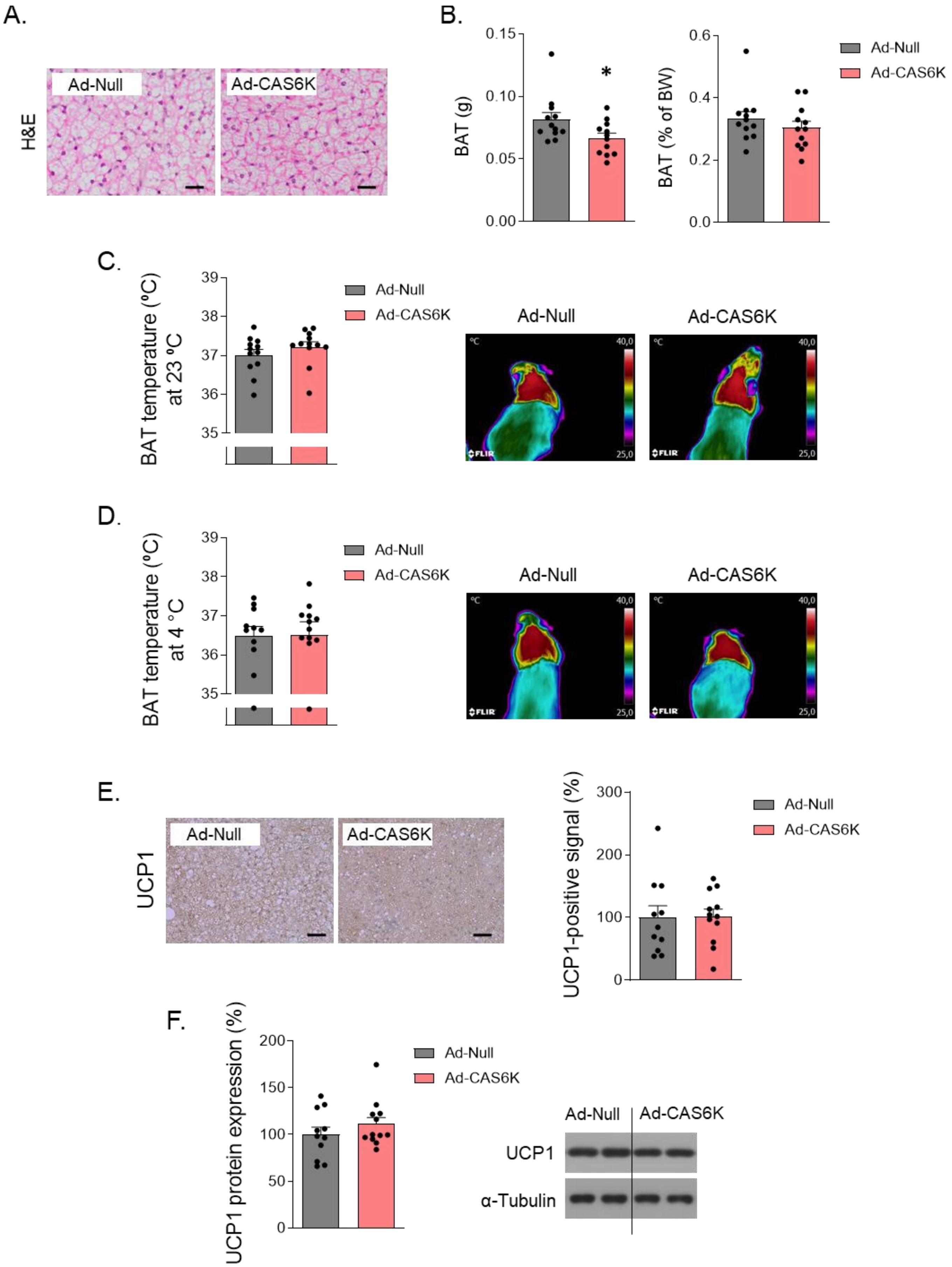

A.
H&E
Ad-Null
Ad-CAS6K
B.
BAT (g)
0.15
0.10
0.05
0.00
*
BAT (% of BW)
0.6
0.4
0.2
0.0
Ad-Null
Ad-CAS6K
C.
BAT temperature (ºC) at 23 ºC
39
38
37
36
35
Ad-Null
Ad-CAS6K
Ad-Null
Ad-CAS6K
D.
BAT temperature (ºC) at 4 ºC
39
38
37
36
35
Ad-Null
Ad-CAS6K
Ad-Null
Ad-CAS6K
E.
UCP1
Ad-Null
Ad-CAS6K
UCP1-positive signal (%)
300
200
100
0
Ad-Null
Ad-CAS6K
F.
UCP1 protein expression (%)
200
150
100
50
0
Ad-Null
Ad-CAS6K
Ad-Null
Ad-CAS6K
UCP1
α-Tubulin

Figure S3. Effects on BAT (brown adipose tissue) thermogenesis after MBH (medio-basal hypothalamus) activation of p70S6K in OVX (ovariectomized) mice. (**A**) Hematoxylin-eosin staining of BAT. Scale bar: 20µm. (**B)** BAT weight expressed in grams or as percentage of BW. (**C–D)**. Representative infrared thermal images and quantification of BAT interscapular temperature (BAT) under basal (**C**) and cold conditions (**D**). (E-F) UCP1 protein levels in BAT determined by (**E**) immunohistochemistry (scale bar: 50 µm) and (F) western blotting. Representative blots are shown. Black line indicates cropped images. Values are expressed as mean ± SEM (n = 12 per group). T-test or Mann-Whitney analyses were used to evaluate statistical differences between Null and CAS6K mice.
